# Pathophisiology of Sickle Cell Disease and New Drugs for the Treatment

**DOI:** 10.4084/MJHID.2009.024

**Published:** 2009-12-20

**Authors:** Lucia De Franceschi

**Affiliations:** Dept of Clinical and Experimental Medicine, University of Verona, Italy

## Abstract

A homozygous mutation in the gene for β globin, a subunit of adult hemoglobin A (HbA), is the proximate cause of sickle cell disease (SCD). Sickle hemoglobin (HbS) shows peculiar biochemical properties, which lead to polymerizing when deoxygenated. HbS polymerization is associated with a reduction in cell ion and water content (cell dehydration), increased red cell density which further accelerate HbS polymerization. Dense, dehydrated erythrocytes are likely to undergo instant polymerization in conditions of mild hypoxia due to their high HbS concentration, and HbS polymers may be formed under normal oxygen pressure. Pathophysiological studies have shown that the dense, dehydrated red cells may play a central role in acute and chronic clinical manifestations of sickle cell disease, in which intravascular sickling in capillaries and small vessels leads to vaso-occlusion and impaired blood flow in a variety of organs and tissue. The persistent membrane damage associated with HbS polymerization also favors the generation of distorted rigid cells and further contributes to vaso-occlusive crisis (VOCs) and cell destruction in the peripheral circulation. These damaged, dense sickle red cells also show a loss of phospholipid asymmetry with externalization of phosphatidylserine (PS), which is believed to play a significant role in promoting macrophage recognition with removal of erythrocytes (erythrophagocytosis). Vaso-occlusive events in the microcirculation result from a complex scenario involving the interactions between different cell types, including dense, dehydrated sickle cells, reticulocytes, abnormally activated endothelial cells, leukocytes, platelets and plasma factors such as cytokine and oxidized pro-inflammatory lipids. Hydroxycarbamide (hydroxyurea) is currently the only drug approved for chronic administration in adult patients with sickle cell disease to prevent acute painful crises and reduce the incidence of transfusion and acute chest crises. Here, we will focus on consolidated and experimental therapeutic strategies for the treatment of sickle cell disease, including:

- agents which reduce or prevent sickle cell dehydration
- agents which reduce sickle cell-endothelial adhesive events
- nitric oxide (NO) or NO-related compounds
- anti-oxidant agents

Correction of the abnormalities ranging from membrane cation transport pathways to red cell-endothelial adhesive events, might constitute new pharmacological targets for treating sickle cell disease.

## Introduction:

A homozygous mutation in the gene for β globin, a subunit of adult hemoglobin A (HbA), is the proximate cause of sickle cell disease (SCD). Sickle hemoglobin (HbS) shows peculiar biochemical properties, which lead to polymerizing when deoxygenated. Studies of the kinetics of HbS polymerization following deoxygenation have shown it to be a high order exponential function of haemoglobin concentration, thus highlighting a crucial role for cellular HbS concentration in sickling^1^,^2^. HbS polymerization is associated with a reduction in cell ion and water content (cell dehydration), increased red cell density which further accelerate HbS polymerization^1^–^3^. Dense, dehydrated erythrocytes are likely to undergo instant polymerization in conditions of mild hypoxia due to their high HbS concentration, and HbS polymers may be formed under normal oxygen pressure.

Pathophysiological studies have shown that the dense, dehydrated red cells may play a central role in acute and chronic clinical manifestations of sickle cell disease, in which intravascular sickling in capillaries and small vessels leads to vaso-occlusion and impaired blood flow in a variety of organs and tissues^2^,^4^. The persistent membrane damage associated with HbS polymerization also favors the generation of^5^ distorted rigid cells and further contributes to vaso-occlusive crisis (VOCs) and cell destruction in the peripheral circulation. These damaged, dense sickle red cells also show a loss of phospholipid asymmetry with externalization of phosphatidylserine (PS), which is believed to play a significant role in promoting macrophage recognition with removal of erythrocytes (erythrophagocytosis), cell apoptosis and activation of coagulation. Although the percentage of dense erythrocytes does not predict the severity of the disease, it has been shown to increase prior to or during the first phase of the painful crisis and to decrease thereafter^4^,^6^,^7^. Vaso-occlusive events in the microcirculation result from a complex scenario involving the interactions between different cell types, including dense, dehydrated sickle cells, reticulocytes, abnormally activated endothelial cells, leukocytes, platelets and plasma factors such as cytokines^8^,^9^ and oxidized pro-inflammatory lipids^6^,^10^,^11^.

Hydroxycarbamide (hydroxyurea) is currently the only drug approved for chronic administration in adult patients with sickle cell disease to prevent acute painful crises and reduce the incidence of transfusion and acute chest crises^12^. Long-term use of hydroxycarbamide has been demonstrated to produce dramatic reductions in mortality and morbidity in patients with sickle cell disease^13^. Clinical use of hydroxycarbamide in pediatric and adult patients with sickle cell disease is discussed in the next chapter on clinical management (13.2). Decitabine has also been shown to be a promising agent for the modulation on Hb F in sickle cell disease^14^. We will focus here on therapeutic strategies currently being considered for the treatment of sickle cell disease, which are not based on Hb F modulation. They include:

- Use of agents which reduce or prevent sickle cell dehydration
- Use of agents which reduce sickle cell-endothelial adhesive events
- Use of nitric oxide (NO) or NO-related compounds
- Use of antioxidant agents

## a) Prevention of sickle red cell dehydration:

One of the distinguishing characteristics of sickle cell disease is the presence of dense erythrocytes, formed as a result of cell dehydration and loss of potassium (K^+^). These dense red cells generally have a lower HbF content and include both reticulocytes and red cells^15^. Usually, the dense fraction of erythrocytes has a high percentage of irreversible sickle cells (ISCs), cells that maintain their sickle shape even when fully oxygenated. An inverse correlation has been demonstrated between percentage of ICSs and erythrocyte survival. In vitro and in vivo studies in animal models for sickle cell disease have suggested a crucial role of dehydrated red cells in the pathogenesis of vaso-occlusive events; in fact, the dense, dehydrated red cells might be easily trapped in post capillary venules, promoting micro-vascular obstruction^16^.

Thus, prevention of red cell dehydration represents an exciting possible new therapeutic strategy. Studies on membrane permeability in sickle cell disease have shown abnormalities in different specialized membrane-embedded transporters that carry cations, anions and water across the erythrocyte membrane. In the last two decades, studies on the nature and properties of the pathways mediating K^+^ loss in sickle cell erythrocytes have led to the development of new therapeutic tools to block K^+^ loss and dehydration.

**Figure 1.**
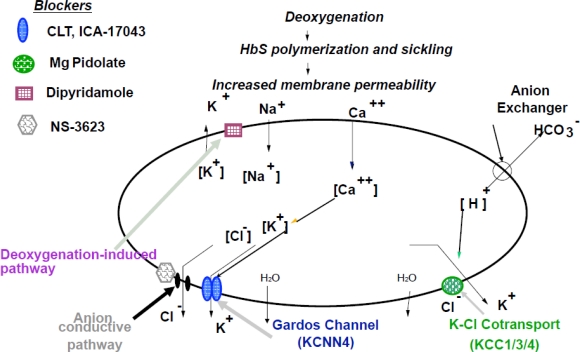
Schematic diagram of the ion transport pathways involved in sickle cell dehydration and action sites of potential therapeutic blockers: *Ca^2+^activated K^+^ channel (Gardos channel, KCNN4):* Clotrimazole (CLT) and ICA-17043; *K-Cl cotransport (KCC1/3/4)*: Magnesium (Mg) Pidolate; *Deoxygenation-induced pathway*: Dipyridamole; *Anion conductive pathway:* NS3623. Deoxygenation induces Hb S polymerization and sickling, with associated increased membrane permeability and abnormal function of different ion transport pathways, resulting in K^+^, Cl^−^ and water loss and red cell dehydration (modified from De Franceschi L et al. Haematologica 89: 348, 2004).

The major pathways for K^+^ loss during sickle cell dehydration events are the Ca^2+^-activated K^+^ channel, known as Gardos channel, operating in parallel with the conductive Cl^−^ pathway and the electroneutral K-Cl cotransport (Figure 1)^17^–^22^.

### Ca^2+^-activated K^+^ channel (Gardos channel, KCNN4):

Sickle red cells are characterized by increased amounts of calcium, which is functionally and physically sequestered into intracellular vesicles, but maintained in normal concentration in the steady state. The cyclic deoxygenation and HbS polymerization that occurs in sickle red cells has been shown to produce transient increase in free intracellular calcium, which is responsible for large K^+^ loss with associated Cl− and water loss. This effect is due to activation of a specific Ca-gated K^+^ channel that was first described by Gardos^23^. The imidazole antimycotic clotrimazole (CLT) has shown to be a specific inhibitor of the Gardos channel and to prevent sickle cell dehydration *in vitro*^18^. In a transgenic mouse model of sickle cell disease, oral administration of CLT was reported to specifically block the Gardos channel, increase the red cell K^+^ content and reduce red cell dehydration^24^. The compound was further tested in normal humans (AA) and in sickle cell volunteers (SS), and was shown to be a powerful and effective inhibitor of the erythroid Gardos channel and of sickle red cell dehydration^25^,^26^. Further studies led to the development of a novel class of compounds based on the back-bone structure of CLT, which have conserved Gardos channel inhibitory power, but are devoid of the imidazole moiety of CLT, and thus of cytochrome P450 inhibitory effects^27^. One of these compounds (ICA-17043) has been shown to have 10-fold greater potency than CLT in blocking the Gardos channel in vitro and in vivo to specifically inhibit Gardos channel and prevent K^+^ loss and red cell dehydration^28^. Phase I studies in normal human subjects and in sickle cell patients, showed significant blockade of the Gardos channel, in absence of any significant side-effects^29^. A phase II study showed that ICA-17043 reduced haemolysis and the percentage of dense cells, with a significant amelioration of anaemia in patients with sickle cell disease^30^. However, a Phase III clinical trial showed no effect of ICA-17043 on the rate of painful events in SCD patients, most likely related with some effects on blood viscosity of red cells displaying an increase survival. No other studies have been planned with this molecule.

Another therapeutic agent, which has been recently shown to modulate the Gardos channel activity, is L-Arginine. Patients with SCD show a state of relative depletion of arginine, which is part of the nitric oxide pathway. L-Arginine supplementation of transgenic sickle cell mice resulted in inhibition of erythrocyte Gardos channel activity and amelioration of red cell dehydration^16^. A phase II study to test the effect of arginine supplementation have shown no major effects on Gardos channel function and erythrocyte hydration in patients with sickle cell disease^31^,^32^.

### K-Cl cotransport (KCC1/3/4):

Several forms of K-Cl cotransport have been described in various human and mouse tissues. KCC2 expression seems to be limited to brain cells, while human and mouse erythrocytes seem to possess KCC1, KCC3 and KCC4 isoforms in different and still undetermined ratio. The K-Cl cotransport mediates red cell dehydration in SCD. Studies on K-Cl cotransport function have identified different triggers of activation, such as cell swelling, cell acidification, reduced cell magnesium (Mg^2+^) content, membrane oxidative damage and urea. Franco et al.^22^ have also shown that K-Cl cotransport mainly contributes to dehydration of sickle reticulocytes and that deoxygenation of sickle red cells also stimulates K-Cl cotransport in isotonic solutions at pH 7.4 (Figure 1). The relative contribution of the Gardos channel and of the K-Cl cotransport pathway in generating dehydrated, dense sickle red cells is a complex and still unresolved issue.

K-Cl cotransport activity is modulated by red cell Mg content and low Mg^2+^ levels are associated with abnormal activation of K-Cl cotransport. Some small studies have reported a reduction in red cell Mg^2+^ content in SCD patients. Thus, oral Mg supplementation with the aim of increasing red cell Mg^2+^ levels and inhibiting K-Cl cotransport activity may represent a possible therapeutic strategy for ameliorating SCD red cell dehydration^16^,^17^. Dietary magnesium supple-mentation in transgenic sickle cell mice has demonstrated that increasing erythrocyte Mg^2+^ content can ameliorate red cell dehydration. Two uncontrolled trials of oral supplementation with Mg pidolate have been carried out in sickle cell patients, showing a reduction in K-Cl cotransport activity, an increase in red cell K^+^ and Mg^2+^ content, an improvement in red cell dehydration and a reduction in the number of painful events^17^,^33^. A double-blind, placebo controlled crossover study with Mg pidolate supplementation in children with sickle cell disease did not demonstrate any significant changes in the haematological parameters studied; however, the Mg-pidolate dosage used was markedly lower than that proposed in the previous studies. In a phase I study, the therapeutic association of Mg-pidolate with hydroxyurea have been evaluated in patients with HbSC disease, showing a significant reduction in the activity of the K-Cl cotransport after 3 months of supplementation^34^.

Recently, it has been reported that infusion of Mg sulfate reduces the length of stay of sickle cell patients hospitalized during vaso-occlusive crises^16^.

### Cl^−^ permeability pathway:

Studies on the conductive Cl− pathway indicate that for red cell dehydration the movement of K^+^ must be accompanied by that of chloride (or other monovalent anions) to maintain electroneutrality (Figure 1). Elegant sets of studies demonstrate that movement of K^+^ and dehydration via the Gardos channel can be blocked if the Cl^−^ conductive pathway is inhibited. A specific inhibitor of Cl-conductance has been recently developed (NS3623). NS3623 has been tested in transgenic sickle cell mice and was found to reduce in vivo sickle cell dehydration, with a mild echinocytosis at the highest doses. Unfortunately, NS3623 was not further developed for clinical use because of undesirable side effects observed in human subjects^16^,^19^.

## b) Anti-adherence therapy in sickle cell disease:

Vaso-occlusive episodes are central events in the pathophysiology of sickle cell disease, causing the clinical manifestations and leading to acute and chronic organ damage. The abnormal adhesive interactions between erythrocyte, reticulocytes, endothelial cells, platelets or soluble mediators may represent a possible new therapeutic target. In addition, SCD patients showed abnormally activated circulating endothelial cells that increase during acute vaso-occlusive crisis suggesting the presence of chronic vascular endothelial damage further worsening during acute events^4^,^35^–^37^. The end-point of anti-adherence therapy is to interfere with the initialization and/or amplification of adhesive events. Although anti-adherence therapy has been mainly studied during acute painful events, its mechanisms of action are only partially known.^4^,^6^,^38^–^40^

**Figure 2.**
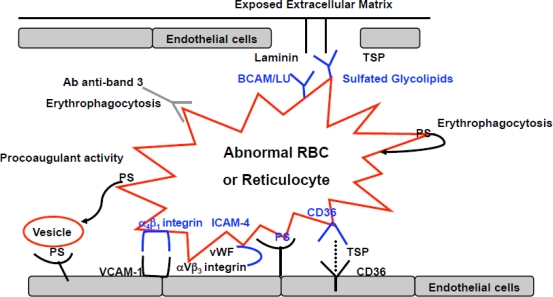
Schematic diagram of possible therapeutic targets for agents that interfere with adherence of sickle red cells (RBC) or reticulocytes to abnormally activated endothelial cells. PS: phosphatidylserine; TSP: thrombospondine; Ab anti-band 3: natural occurring antibodies (NTAb) anti-band 3; vW: von Willebrand; BCAM/LU: Lutheran blood group protein; ICAM-4: Landstein-Weiner (LW) blood group glycoprotein (modified from De Franceschi L et al. Haematologica 89: 348, 2004).

In SCD, the anti-adherence therapeutic strategies (Figure 2) can be divided into:

- Molecules interfering with chemical-physical processes during erythrocyte-endothelial adhesion events
- Molecules interfering with sickle cell-endothelial adhesive mechanisms
- Molecules modulating inflammatory pathways involved in sickle cell-endothelial adhesion
- The heme-oxygenase-1 (HO-1) connection

### Molecules interfering with chemical-physical processes during erythrocyte-endothelial adhesion events:

Non-ionic surfactant block copolymer such as RheothRx (Poloxamer 188) lowering viscosity and frictional forces improves microvascular blood flow. RheothRx has been shown to block hydrophobic adhesive interactions (cell-cell, cell-protein or protein-protein interaction) in blood, resulting in reduction of erythrocyte aggregation and red cell adherence to vascular endothelium, with a hypothetical improvement in microvascular flow^41^. Phase II studies have shown a limited favorable effect in treatment of acute pain crises, when associated with hydroxyurea (HU) in sickle cell children. However, no further clinical development studies are planned for this compound.

### Molecules interfering with sickle cell-endothelial adhesive mechanisms:

Recent studies on the sickle cell-endothelium adhesive mechanism have identified different interactions which may have particular therapeutic relevance: a) the integrin α a4bβ1 receptor of fibronectin and the vascular adhesion molecule −1 (VCAM-1), E-selectin and P-selectin; b) the thrombospondin and/or collagen and receptor CD36, present on the surface of endothelial cells, platelets and reticulocyte-rich subpopulations of normal and sickle erythrocytes; c) the sulfate glycolipids, which bind thrombospondin, von Willebrand factor multimer and laminin^2^,^42^,^43^; d) the Lutheran blood group proteins (BCAM/LU), which expression is increased in red cells from SCD patients that bind to α5 subunit of laminin, a component of extracellular subendothelial matrix^44^,^45^; e) the ICAM-4 (Landstein-Weiner blood group glycoprotein- LW), which binds αVβ3 integrin receptors on endothelial cells^46^–^49^; and f) the exposure of phosphatydyl-serine (PS), detectable in a subpopulation of sickle red cells, which participates in sickle cell adhesion to activated endothelium^50^–^54^ (Figure 2). *Ex vivo* and *in vitro* experimental studies have shown that thrombospondin- and von Willebrand factor-mediated interaction between sickle red cells and endothelium via α Vβ3 integrin might be blocked by monoclonal antibodies against αVβ3 integrin receptors^42^,^48^,^55^. Recent study with short synthetic peptides interfering with ICAM-4 and α Vβ3 integrin binding have been evaluated in ex vivo system, showing a reduction in sickle erythrocyte adhesion to activated endothelial cells allowing to consider the blocking of this adhesion pathway as possible therapeutic new strategy in treatment of acute sickle cell events^48^,^55^.

The binding between thrombospondin, von Willebrand factor and laminin, which mediates sickle cell-endothelial adherence, might be blocked by anionic polysaccharides such as high molecular weight dextran sulfate or chondroitin sulfate^2^,^42^, ^43^.

An additional therapeutic approach to block sickle cell adhesion to endothelial cells is heparin that might interfer with sickle cell adhesion to endothelial cells through P-selectin^56^–^59^ or binding to TSP that can mediate the interactions between sickle erythrocytes and the vascular endothelial surface. A double blind randomized trial with tinzaparin in SCD patients during acute VOCs has documented a reduction of severity and duration of the VOCs^51^,^60^.

### Molecules modulating inflammatory pathways involved in sickle cell-endothelial adhesion:

Chronic inflammatory state has been described in SCD patients characterized by increase plasma levels of acute phase proteins, of soluble cytokines such as IL1β, IL6, TNF- α and endothelin-1 (ET-1) that are further elevated during acute VOCs. These factors participate to leukocyte chemotasis, modulate vascular tone and contribute in sickle cell related tissue damage. Thus, anti-inflammatory therapy has been propose to interfere with inflammatory storm and abnormal vascular activation^61^. Sulfasalazine is an anti-inflammatory molecule and can inhibit the transcription of nuclear factor NF-kB and interferring with endothelial cell activation^62^–^65^. Transgenic sickle cell mice treated with sulfasalazine show a reduction in activated circulating endothelial cells, and in VCAM-1, ICAM and E-selectin vascular wall endothelial expression. In a pilot study, the administration of sulfasalazine to sickle cell patients results in reduction in the abnormal endothelial activation^4^. Another possible strategy aimed at reducing the adhesion of sickle red cells to vascular endothelium is the inhibition of interactions between leukocytes already adherent to endothelium and sickle red cells during vaso-occlusive events^66^. Based on the *in vitro* evidence that immunoglobulin (Ig) significantly reduces the binding of sickle red cells to neutrophils in transgenic sickle cell mice, the infusion of Ig in vivo was shown to inhibit the interaction between sickle red cells and leukocytes in the cremasteric venules, suggesting that Ig may act either by inhibitiing the interactions between sickle red cells and leukocytes and/or by reducing the number of adherent leukocytes^66^. In humans, three out of four sickle cell patients treated with infusion of Ig showed some beneficial effect, whereas in the fourth case the treatment accelerated a vaso-occlusive crisis^66^,^67^. Since Ig infusion might be related to severe side effects such as renal toxicity and thrombosis, it should be used with caution in sickle cell patients.

Recent studies have shown the important role of ET-1 in acute sickle cell related VOCs in a mouse model for SCD^68^,^69^. The block of ET-1 actions was obtained directly by the ET-1 receptors antagonist, Bosentan, evaluating its effects on SCD mouse kidney as target organ and indirectly by the inhibition of phosphodiesterase-4 with Rolipram in a model of early pulmonary hypertension^68^,^69^. Bosentan is actually under evaluation in a phase III clinical trial in SCD patients with pulmonary hypertension^70^.

The possibility of delivering oxygen directly to sickled red cells entrapped in partially obstructed vessels has also been explored. Perflubron-based fluorocarbon emulsion (PFE) decreases the peripheral vascular resistance ex vivo in the mesocaecal vasculature of rats, due more to its ability to dissolve oxygen than to its ability to modify the vascular tone^71^.

Recently, Hebbel et al have reported the beneficial effects of histone deacetylase inhibitors on vascular pathology in mouse model for sickle cell disease^72^. The Authors observed multiple therapeutic effects of these compounds as: (i) inducers of HbF; (ii) iron chelators; (iii) modulators of vascular damage and abnormal activation sickle cell related (i.e.: reduction of VCAM-1 expression).

### The heme-oxygenase-1 (HO-1) connection:

In different sickle cell mouse models under steady state, Belcher et al have shown up-regulation of cytoprotective gene as heme oxygenase-1 (HO-1) and a reduction of the sickle cell related organ damage when pathological mice were treated with heme oxygenase-1 products^63^,^73^. In SCD patients in steady state, the gene expression profiling of circulating leukocytes has shown increased HO-1 and biliverdin reductase as well as in kidney and in circulating endothelial cells^74^,^75^, suggesting an induction of cell protective systems in response to chronic inflammatory stress characterizing SCD. However, the still open question is whether these cytoprotective systems are rapidly and further induced driving a continuous cellular protection during acute sickle cell vaso-occlusive crisis.

## c) Nitric Oxide (NO) based therapies in sickle cell disease:

Nitric oxide (NO) is a potent vasodilator and inhibitor of vascular remodeling and also affects the multi-step cascade of events involved in leukocyte, platelet and endothelial activation. NO is generated from L-Arginine by endothelial cells via constitutive (eNOS) and inflammatory inducible nitric oxide synthases (iNOS). SCD is characterized by relative reduction in NO bioavaibility that contributes to endothelial abnormal activation and SCD organ damage. In addition chronic hemolysis leading to increase the plasma levels of hemoglobin that is an efficient NO buffer, contributes in reducing NO levels in SCD.

Recent studies have focused on inhaled NO for the treatment of tissue damage in various ischaemic syndromes, including cardiovascular disease, pulmonary hypertension, and acute lung distress syndromes. The possible therapeutic role of inhaled NO has been studied in different animal models of lung injury induced by ischaemia/reperfusion. Inhaled NO prevents leukocyte migration and reduces the permeability of the peripheral microvasculature. In association with surfactant, inhaled NO alleviates alveolar edema and reduces bronchoalveolar leukocyte and neutrophil infiltration in animal models of ischaemic lung injury. A placebo-controlled randomized clinical trial of inhaled NO in SCD has recently reported beneficial results in the treatment of acute vaso-occlusive crisis, although the mechanism of action remains unknown. Plasma NO metabolites are decreased in SCD patients during vaso-occlusive crisis associated with severe pain and also in acute chest syndrome^32^. A decrease in exhaled NO has been reported in sickle cell patients, suggesting a role for NO in the pathogenesis of the pulmonary complications^76^. In a transgenic mouse model of sickle cell disease, it has been shown that inhaled NO provides protection during ischaemia/reperfusion lung injury, in which endothelial NO production is reduced^77^,^78^.

In addition NO-donor as polynitroxy-albumine and nitroxic- albumine have been shown to be effective in reducing inflammatory state in a SCD mouse strain and to reduce the hypoxia induce lung damage in another mouse model of acute VOCs^79^,^80^.

Another possible therapeutic strategy for increased NO production in sickle cell disease is supplementation of L-Arginine. Morris et al showed that L-Arginine supplementation alone induces an unexpected decrease in NO metabolite production^11^,^81^. In a subsequent pilot study, an increase in NO metabolites was observed when L-Arginine was co-administrated with HU, suggesting that the combination treatment may have a synergistic effect on NO production^2^,^31^. A phase II trial on L-Arginine supplementation in SCD has shown no effects on NO levels and on erythrocyte features.

## d) Antioxidant agents in sickle cell disease:

SCD is characterized by a pro-oxidant environment due to high production of reactive oxygen species (ROS) related to increased levels of free pathological iron and heme groups associated with a reduction in antioxidant systems such as GSH^82^–^85^. Studies *in vitro* on SCD red cells have shown that iron chelation by deferipone (L1) reduce the sickle red cell membrane susceptibility to iron mediated oxidative damage^82^,^86^. In vivo study on SCD patients supplemented with L-glutamate to increase GSH and glutamate levels have shown some improvement of chronic pain^83^.

## Conclusions:

In conclusion, the emerging picture for treatment of sickle cell disease is that abnormalities ranging from membrane cation transport pathways to red cell membrane proteins structure and function, or red cell-endothelial adhesive events, might constitute new pharmacological targets for treating sickle cell disease. Prospective therapy for SCD need to combine molecules with different pharmacological targets in order to increase their therapeutic efficacy and to reduce their side effects (e.g., volume-controlling drugs and either hydroxyurea or anti-adhesive molecules).
